# A Survey of Commercial Pine Turpentine Essential Oil Products from the Turkish Market and Their Compliance with European Pharmacopoeia 10.0

**DOI:** 10.3390/molecules31040737

**Published:** 2026-02-21

**Authors:** Tuğba Buse Şentürk, Nehir Kavi, Timur Hakan Barak, Engin Celep

**Affiliations:** 1 Department of Pharmacognosy, Faculty of Pharmacy, Acibadem Mehmet Ali Aydinlar University, Istanbul 34752, Türkiye; tugba.avci@acibadem.edu.tr (T.B.Ş.); nehir.kavi@acibadem.edu.tr (N.K.); engin.celep@acibadem.edu.tr (E.C.); 2 Institute of Graduate Studies in Health Sciences, Istanbul University, Istanbul 34126, Türkiye

**Keywords:** pine turpentine, essential oil, *Pinus pinaster* Ait., GC-MS, European Pharmacopoeia

## Abstract

Pine turpentine essential oil (PTEO) obtained from *Pinus pinaster* Ait. is known to be used in many fields such as medicine, cosmetics, agriculture, and art. The medicinal use of essential oils puts pressure on industry to produce high-quality products. Pure essential oils derived from natural sources are mistakenly recognized as safe on the grounds of their natural origin. Unless they meet international standards, their safety remains questionable. Therefore, in this study, it was aimed to evaluate the quality of 14 different pine turpentine essential oil samples purchased from various sources on the Turkish market, based on the legally recognized pharmacopoeia in Türkiye, the European Pharmacopoeia (EP). As stated in the “Turpentine Oil” monograph, appearance, relative density, refractive index, optical rotation, acid value, peroxide value, fatty oils, and resinified essential oils analyses were performed for each sample. Additionally, phytochemical profiles were analyzed by high performance thin-layer chromatography (HPTLC) and gas chromatography-mass spectrometry (GC-MS). The results revealed that none of the samples were compliant with EP standards. With this in mind, it is found necessary to impose strict regulations on the production of commercial essential oils. Nevertheless, pharmacies emerge as preferable options for obtaining such products.

## 1. Introduction

As the growing interest in natural treatments steadily grows, the cosmetic and medicinal use of essential oils is correspondingly increasing. Consequently, scientific studies on the role of essential oils in treatment are also on the rise [1]. The genus *Pinus* is the largest species in the Pinaceae family and consists of about 115 species of conifers and evergreen trees [2]. The recorded number of *Pinus* species in Türkiye is known to be five (*P. brutia*, *P. nigra*, *P. sylvestris*, *P. pinea*, and *P. halepensis*) [3]. Although *P. pinaster* is not native to Türkiye and does not occur naturally in its flora, the Black Sea, Marmara, and Aegean Sea coasts are exceptions in present conditions for bearing this species [4]. Yet, its natural presence in the Ankara region during the Miocene period is proven with fossil records [5].

Pine tree bark contains oleoresins, which are composed of essential oil and resin. Oleoresins are collected by wounding the outer layer of the bark and tapping them. Next, oleoresin is distilled to obtain the pine turpentine essential oil (PTEO). It consists of approximately 70–85% α-Pinene, and its odor is strong and bitter [6]. PTEO plays an important role for the cosmetics and food industry with its fragrance or additive essence properties. It is also used in aromatherapy as emmenagogue, carminative, and rubefacient agents. The chemical differences of these essential oils arise from ecological, seasonal, and genotypic reasons and various reasons related to their preservation [7]. Pharmacopoeias contain the standard values of natural-origin medicinal products and synthetic substances used in the production of drugs in order to fulfill quality requirements. Türkiye is officially affiliated with the European Pharmacopoeia (EP) to ensure compliance with international standards [8]. In Türkiye, various essential oils are widely used, including PTEO, among the public for both cosmetic and medical purposes. There are many natural products on the Turkish market that claim to contain pure essential oils according to their labels. Due to essential oils being highly sensitive to factors such as plant origin, harvesting conditions, extraction methods, and storage, it is of great importance to evaluate the quality standards of the products in the market according to the European Pharmacopoeia. Moreover, without proper quality assessment, issues such as adulteration, contamination, oxidation, or degradation may not be prevented. Since these products may be used for therapeutical purposes, failure to comply with EP standards poses a potential risk to public health, as non-compliant essential oils may lack their expected biological activity and may lead to allergic reactions or other undesirable adverse effects. Therefore, standardization according to EP guidelines has become necessary to ensure the safety and efficacy of essential oils marketed for human use. Despite the abundance of studies in literature evaluating the quality of essential oils on the market, no specific research has been conducted on PTEO based solely on EP criteria.

In this study, pine turpentine essential oil samples from 14 different brands that claim to be pure according to the label were examined. At the time the analyses were conducted, the European Pharmacopoeia (10th edition) was the current and officially applicable version in the region. Specimens were obtained from various sources such as the internet, herbalists, and pharmacies. In order to evaluate the difference between sales places, 4 samples were obtained from pharmacies, and the remaining 10 samples were obtained from different sales points other than pharmacies. The appearance test, high performance thin-layer chromatography (HPTLC) analysis, fatty oils, and resinified essential oils tests showed visible results, whereas relative density, refractive index, optical rotation, acid value, and peroxide value tests were evaluated with numerical data for each sample. In addition to that, gas chromatography-mass spectrometry (GC-MS) analysis was applied to all samples to examine the phytochemical profile of essential oils. The European Pharmacopoeia monograph on “Turpentine Oil” defines the optimal range for nine chemical components with their percentage amounts. The results of GC-MS analyses were compared with these criteria. This study aimed to evaluate the current quality status of PTEO available on the Turkish market. For public health protection, it is crucial to conduct these analyses on medicinal products to ensure compliance with international standards.

## 2. Results

### 2.1. Appearance

**Figure 1:**
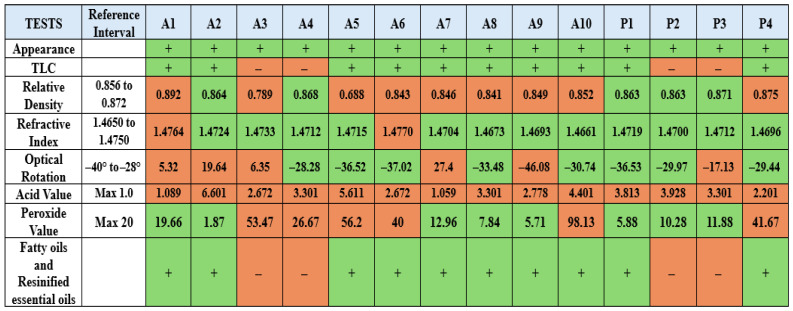
Overall results table. Green represents compliance with the EP and orange represents non-compliance.

According to the criteria defined in the European Pharmacopoeia 10.0, PTEO should have the appearance of a clear, colorless, or pale-yellow liquid. The suitability of the samples according to the criteria is shown in Figure 1.

### 2.2. Relative Density, Refractive Index, Optical Rotation, Acid Value, and Peroxide Value

The results of relative density, refractive index, and optical rotation tests were given in Table 1. Acid value and peroxide value test results were given in Figure 1. To comply with the EP, the relative density must fall within the range of 0.856 to 0.872, the refractive index within the range of 1.4650 to 1.4750, and the optical rotation within the range of −40° to −28°. The acid value should be a maximum of 1.0, and the peroxide value should be a maximum of 20. Their compliance with the European Pharmacopoeia has been presented in Figure 1.

### 2.3. HPTLC Analysis, Fatty Oils, and Resinified Essential Oils

**Figure 2:**
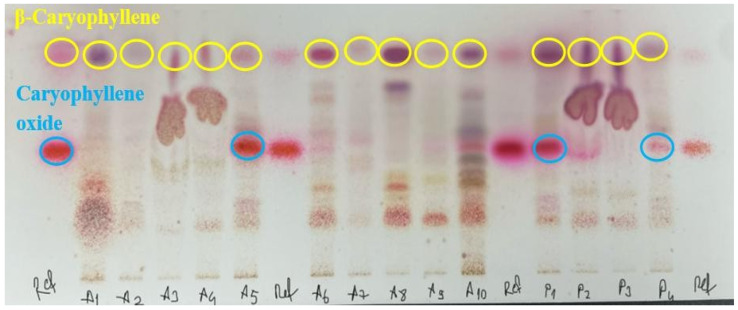
The results of HPTLC analysis for all samples. Ref: Reference mixture. Spots marked with a yellow circle are β-Caryophyllene; spots marked with blue circles are caryophyllene oxide.

**Figure 3:**
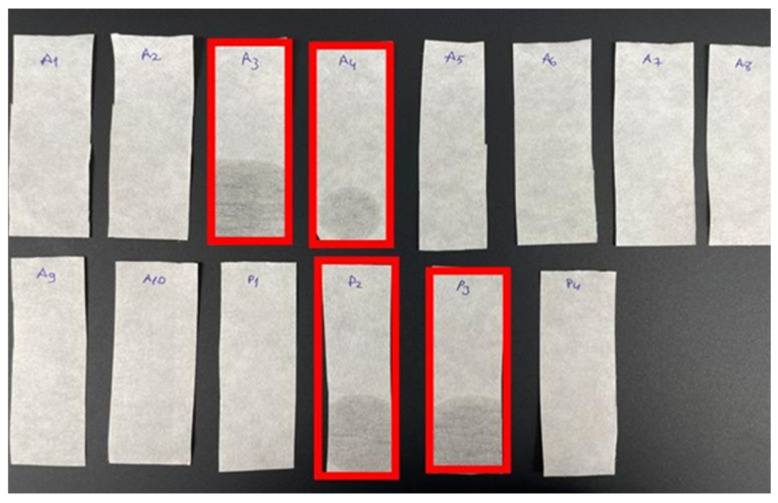
Fatty oils and resinified essential oil assay results. Samples in the red rectangle were noncompliant with EP.

According to the European Pharmacopoeia, β-caryophyllene, one of the nine chemical ingredients, should form a pink spot on the upper part of the silica gel plate. Likewise, caryophyllene oxide should form a pink spot in the middle part of the plate. Also, a brownish-purple spot should be present on the lower parts of the plate, where the test solution was applied. The results of the analyses of 14 samples were given in Figure 2. The compatibility of the samples with the pharmacopoeia can be observed in Figure 1. According to the EP criteria set for the fatty oils and resinified essential oils test, essential oils should not leave stains after evaporation. Sample compatibility can be seen in Figure 3.

### 2.4. GC-MS Analysis

**Figure 4:**
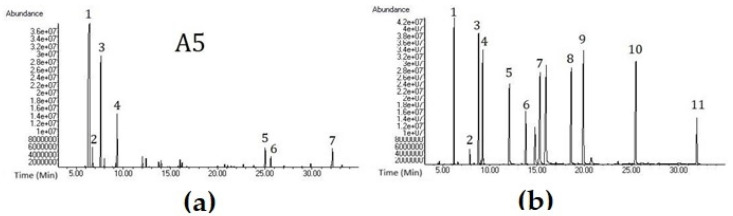
(**a**) GC-MS chromatogram of A5 sample. 1: α-Pinene, 2: Camphene, 3: β-Pinene, 4: Limonene, 5: Longifolene, 6: β-Caryophyllene, 7: Caryophyllene oxide; (**b**) GC-MS chromatogram of standard mixture. 1: α-Pinene, 2: Myrcene, 3: α-Terpinene, 4: Limonene, 5: Camphor, 6: endo-Borneol, 7: Terpinen-4-ol, 8: α-Terpineol, 9: Bornyl acetate, 10: β-Caryophyllene, 11: Caryophyllene oxide.

**Figure 5:**
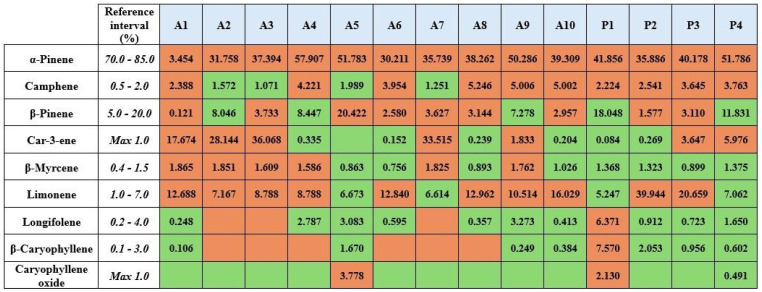
Results of the GC-MS analyses. Green represents compliance with the EP; orange represents noncompliance.

The phytochemical profile of a total of 14 samples was analyzed using GC-MS, and the results are listed in Table 2. A total of 47 different chemical components were identified. At least 90.6% and at most 99.7% of the PTEO chemical profile were resolved. A representative chromatogram is shown in Figure 4 for illustration of the 9 phytochemical ingredients specified in the EP. A reference/standard mixture was prepared and analyzed to confirm the results. This reference mixture contained selected chemical components that were found in phytochemical profile analysis of the samples (Figure 4). The objective was to create an essential oil standard library in order to enhance the reliability of the analysis. The compliance of GC-MS analyses results with the pharmacopoeia is given in Figure 5.

## 3. Discussion

Approximately 12% of the EP is devoted to plant-based monographs. These monographs include herbal drugs and herbal medicine preparations, which are herbal teas, extracts, and essential oils [9]. Given their medicinal and cosmetic significance, highlighting the critical gap in the market regarding the authenticity and quality of commercially available essential oil-containing products was considered a necessity. In addition to this study, the findings of other studies support the significant difference between label claims and actual product composition. Therefore, focusing on this subject as a contribution to growing awareness was the primary goal to show the possible systemic challenges of the essential oil industry. This accumulated data plays an important role in attracting the attention of healthcare professionals, authorities, and industry. Determining the problem and performing analytical assays might be regarded as the first step of gathering evidence for further solutions. EP herbal monographs are regarded as a reference to perform quality control analyses. These monographs include international standards to determine the quality control of essential oil samples. Each monograph begins with the description of the appearance of the essential oil and provides a list of phytochemicals expected to be present in the mentioned essential oil and specifies their concentration limits in the relevant section.

Today, as the claimed benefits of herbal products are spreading rapidly around the world, a noticeable rise in their utilization is observed. However, this high supply-demand situation can lead to a potential threat, adulteration. Driven mostly by economic aspects, the adulteration of botanical products occurs when supply shortages tempt manufacturers to mix or replace the authentic materials with cheaper alternatives spent to maximize profits by deceiving analytical methods. For essential oils, diluting the authentic oil with a lower-cost essential oil, adding synthetic compounds, and adding “spent” materials such as byproducts and waste products are common adulteration schemes [10]. Therefore, quality control analysis plays an important role in determining the safe use [11]. Lack of studies on compliance with the EP in the literature is noticeable. In a previous study, quality control analysis was carried out according to the EP for 15 different rosemary essential oil samples collected from different points of sale on the Turkish market. The result of this study indicated that none of the samples fully met the criteria. However, the same study has demonstrated that products obtained from pharmacies exhibit more acceptable results compared to samples purchased from other sources [12]. In another similar study, 16 different eucalyptus essential oil samples were obtained from different sources on the market, and meeting the EP standards was evaluated. Similarly, it has been shown that no sample fully met the standards, and products purchased from pharmacies yield more acceptable results than those purchased from herbalists, websites, and beauty stores [13]. In this study, 14 pine turpentine essential oil samples were obtained from different brands. Before conducting the analysis, each sample was examined to verify whether its label claimed ‘100% pure essential oil’. The term ‘pure essential oils’ is frequently used as a marketing tool across various products; however, the high cost of natural ingredients compared to synthetic alternatives has led to widespread adulteration practices. Common methods of adulteration include the addition of lower-cost essential oils or synthetic solvents to maximize profit. Although the safety of essential oils has been extensively studied, the presence of undeclared adulterants may pose unforeseen health risks. Firstly, all samples were analyzed for fatty oils and resinified essential oils tests. The fatty oils and resinified essential oils test is crucial in essential oil analysis, as it provides important insight into the product’s purity and overall quality. As essential oils consist of volatile compounds, they are expected to evaporate almost entirely under appropriate conditions.

The appearance of visible stains may indicate the presence of impurities or production defects, which could compromise the overall quality of the product. This simple test serves as a practical indicator of purity and proper manufacturing practices. Four samples coded A3, A4, and P2, P3 failed the test as a result of leaving stains after waiting for a specified amount of time (Figure 3). Similarly, the same samples failed the HPTLC test by showing distorted spots (Figure 2). These two test results indicated adulteration in samples coded A3, A4, and P2, and P3. Due to the analytical procedure used for fatty oils and resinified essential oils showing minor methodological differences from the European Pharmacopoeia monograph, complete verification of compliance with the EP criteria could not be fully assessed. In the HPTLC test, β-caryophyllene was observed in all samples. However, caryophyllene oxide was only observed in samples coded A5, P1, and P4. In order to find out the required amounts of phytochemicals mentioned, the GC-MS results section of the monograph was consulted. According to the monograph, caryophyllene oxide should be present at a maximum of 1%. However, the absence of caryophyllene oxide on the HPTLC plate does not indicate a negative test result. Even though a pink zone was observed for A10 and P2 coded samples on the HPTLC plate, GC-MS analysis clearly shows no presence of caryophyllene oxide. This might be due to another substance present exhibiting a similar color in the relevant area. Among the quantitative analyses of monograph tests, relative density, refractive index, and optical rotation results are important for assessing substance purity. 9 samples, coded A1, A3, A5, A6, A7, A8, A9, A10, and P4, fell outside the specified value range for the relative density assay. Samples A1 and A6 were not within the specified range for the refractive index assay. In addition to that, specimens A1, A2, A3, A7, and P3 showed values beyond the limits of the monograph for the optical rotation assay. The discrepancies between these assays are linked, as these parameters are highly sensitive to chemical profile and physical properties. However, essential oils may retain their color, as well as some of their physical and chemical properties, including refractive index and relative density—even when mixed with fixed oils. Therefore, relying solely on parameters such as refractive index, relative density, or optical rotation might be insufficient for detecting adulteration, and these should be complemented with additional analytical methods [14]. The acid value measures the alkaline amount required to neutralize one gram of the substance. For oils, a high acidity value is undesirable as it can affect the quality. None of the 14 samples passed this test. The peroxide value measures the amount of peroxide in the sample. Since peroxide is formed as a result of oxidation of oils, a high peroxide value indicates that the samples were not fresh and have started to deteriorate. A3, A4, A5, A6, and A10 were not in compliance (Figure 1).

Gas chromatography-mass spectrometry (GC-MS) is one of the key analytical techniques used to elucidate the chemical composition of essential oils. Since EP emphasizes the use of FID as a detector, the results were interpreted upon the requirements of the monograph accordingly. Given that the phytochemical profile directly reflects the compounds responsible for the biological activity of essential oils, its accurate interpretation is essential. The identification of specific components via gas chromatography, as well as the quantitative determination of characteristic marker compounds, is particularly important, as it reflects an approach to assessing the quality of essential oils both as an independent finished product and as part of prophylactic or therapeutic products [15]. Similar tests were conducted in order to visualize the phytochemical profile of different essential oils. Commercial essential oils of *Boswellia carteri*, *B. serrata*, and two chemotypes of *Canarium luzonicum* were analyzed with GC-MS for their phytochemical profile analyses [16]. Chemical components of *Pinus nigra* Arnold species from Türkiye were studied to observe the effect of the seasons and the location on their essential oil content. Results showed that the highest essential oil yield was obtained from the materials that were collected in summertime. Additionally, major component α-pinene was found in higher amounts in the essential oils that were obtained from southern regions of Türkiye [17]. Moreover, there are various studies in the literature that have investigated phytochemical profiles of *Pinus pinaster* oils. In a study, it was determined that *Pinus pinaster* essential oil contains 0.8% α-pinene [18], while another study showed 80.95% presence [19]. Likewise, another important ingredient δ-3-Carene, shows significant variations in the literature in different studies. Rodrigues et al. (2017) showed that different *Pinus pinaster* samples contain variable δ-3-Carene, from trace amounts to 21.8% [20].

Within the aspects of previous studies in order to reveal phytochemical profiles, GC-MS analyses were applied separately for 14 different PTEO samples. More than 90% of the sample ingredients were revealed. However, none of the samples followed the standards of the EP 10.0. According to the ranges specified in the monograph, the major element of PTEO is α-pinene, which should be between 70% and 85%. Unfortunately, none of the 14 samples is close to the limit interval (Figure 5). In addition to that, the A1 coded sample contains 3% α-pinene, demonstrating a serious inaccuracy. A5 and P4 coded samples are the most compliant samples for GC-MS analysis due to containing 6 out of 9 contents with accurate limits (Figure 5). A notable example in the figure is the A3 coded sample, which contains 36% δ-3-Carene, far exceeding the maximum of 1%. It has been stated that in cold regions such as Finland, the amount of δ-3-Carene in PTEO can reach up to 30–40%, yet in warm regions this amount is expected to be lower [21]. Sandaracopimaral, a typical diterpenoid, was detected in P3 and A8 coded samples. In pine oleoresin, monoterpenes and sesquiterpenes are usually found in the volatile fraction, whereas diterpenic acids remain in the non-volatile fraction [22]. Due to poor rectification, PTEO may contain such diterpenoids [23]. Hence, this gives an opinion about the various phytochemical profiles of essential oils in the literature. Yet, the general failure of quality tests enlightens the bigger picture. Adulteration continues to be a major problem in essential oils [24]. A recent study mentioned some novel adulteration detecting techniques such as isotope ratio mass spectrometry (IRMS) [25], Raman spectroscopy [26], chiral gas chromatography [27], and mid-infrared spectroscopy [28]. These promising novel methods can detect adulteration more clearly for further investigations. In a study about detecting adulteration, chiral gas chromatography with a flame ionization detector (GC-FID) is used. Primary essential oils of *Pinus sylvestris* L. showed enantiomeric excess of (+)-α-pinene; however, commercial essential oils with *Pinus sylvestris* L. labels showed enantiomeric excess of (−)-α-pinene similarly in turpentine oil [26]. Economic factors in essential oil production contribute to the adulteration or dilution of pine needle essential oils with turpentine oil due to their similar phytochemical profiles [29]. Given the difference in place of purchase, pharmacy products exhibited more compliant rates to the European Pharmacopoeia compared to other sources (59% and 49.5%, respectively). These rates were calculated for each sample first, based on the number of passed tests divided by the total test numbers, and then this value is calculated as a percentage. This step is continued for each sample. Next, the average of these percentages was calculated for P-coded samples and A-coded samples separately. Essential oils sold freely in uncontrolled environments such as non-pharmacy outlets, herbal shops, cosmetic stores, and online platforms pose a risk of introducing low-quality and substandard products into the market. Compliance with pharmacopoeial standards enhances product reliability and safeguards consumer health. Moreover, companies that analyze and certify their products according to these standards contribute to market order and transparency. Pharmacopoeia monographs and standards also establish definitive quality criteria by regulating the inherent variability in the chemical composition of essential oils caused by multiple factors, thereby providing a trustworthy reference framework for their therapeutic application.

## 4. Materials and Methods

### 4.1. Materials

Samples that claimed to be obtained from *Pinus pinaster* Ait. trees and contain 100% pure PTEO according to their labels were obtained from herbalists, the internet, and pharmacies. The records of all samples in the cosmetic product category were checked in the Ministry of Health of the Republic of Türkiye database. Until the analysis, all samples were stored in a closed box away from daylight. Samples obtained from community pharmacies are given the P code, and samples taken from other sources are given the A code. All standards are sourced from Sigma-Aldrich (St. Louis, MA, USA).

### 4.2. Appearance

All tests were carried out in accordance with European Pharmacopoeia 10.0. It has been implemented with minor changes compared to the 10.0 Edition. In the appearance test, an equal amount of essential oil was taken for each one of the 14 samples and put into the test tubes, respectively. Each test tube was labeled according to sample codes. The color and the appearance of each sample were observed to check the compliance with EP 10.0 criteria. In the fatty oils and resinified essential oils assay, one drop from each sample was dripped onto filter paper and kept in an oven at 100 °C for 10 min (Figure 3) [30].

### 4.3. Relative Density, Refractive Index, Optical Rotation, Acid Value, Peroxide Value

Relative density assay was performed in triplicate by measuring the weight of 1 mL essential oil at room temperature and comparing it to a known sample (water) density. Refractive index assay was performed in triplicate with the Anton Paar (Graz, Austria) -Abbemat 3100 refractometer device. The temperature was adjusted to 20 °C, and we waited until it was set. The magnetic handle was opened, and 2 drops of sample were dropped onto the sample compartment with the help of a Pasteur pipette. Then, the measurement was started. Results were recorded, and the mean value was calculated. Optical rotation assay was performed in triplicate with the Anton Paar (Graz, Austria)—MCP device. Each sample preparation was diluted up to 20 mL with 70% ethanol to achieve a 0.1 g/mL ratio. Before starting the measurement, distilled water was injected into the device compartment to avoid any air bubbles with a syringe, as well as injecting alcohol between the samples to remove any sample residue in the compartment. Obtained results were used in the relevant formula with slight modifications to calculate the optical rotation value. Their mean value was calculated.

[α]tλ = (1000α)/(l × c)

α = angle of rotation measured at temperature t and wavelength λ, in degrees;

l = path length of the polarimeter sample cell, in decimeters;

c = concentration of the solution, in grams per liter.

Acid value assay was performed once, according to EP 2.5.1. subsection with slight modifications under the “Acid Value” title. 1 g of PTEO samples was dissolved in 50 mL of a petroleum ether and 96% ethanol mixture, then titrated with 0.1 M NaOH. Phenolphthalein was used as an indicator. The formula below was used to calculate acid value.

IA = 5.611 n/m

n = volume in mL of used titrant

m = amount of sample used in grams

Peroxide value assay was performed once, accordingly with EP subsection 2.5.5. under the “Peroxide Value” title with slight modifications. A 1 g sample was mixed with 3 mL CH_3_COOH:CH_3_Cl (3:2) *v*/*v* first and then mixed with 0.1 mL saturated KI solution. 3 mL distilled water was added, and the flask was shaken for 1 min. A 1% starch solution was added as an indicator. Next, the prepared solution was titrated with 0.01 N sodium thiosulfate (Na_2_S_2_O_3_). The titration was stopped once the black color turned into white. The initial and final volumes were recorded and used in the formula down below.

IP = (V × N × 100)/WS

V = volume of sodium thiosulfate (mL)

N = normality of sodium thiosulfate

WS = sample weight (g)

### 4.4. HPTLC Analysis, Fatty Oils, and Resinified Essential Oils

Thin layer chromatography was applied according to the instructions in the monograph “Turpentine Oil” in European Pharmacopoeia 10.0 Edition with adaptations in order to use CAMAG HPTLC Automatic TLC Sampler 4 ATS 4 and CAMAG HPTLC Automatic Developing Chamber ADC 2 [24]. The standard solution of HPTLC was prepared by mixing β-caryophyllene and caryophyllene oxide standards from Sigma-Aldrich and dissolving in toluene R. The mobile phase was prepared with ethyl acetate R and toluene R (5:95 *v*/*v*). A silica gel F254 plate was used for the stationary phase. The plate was sprayed with anisaldehyde solution R, and it was put in the oven at 100–105 °C, and we waited for 5 min. The plate was examined under daylight.

In the fatty oils and resinified essential oils assay, slight modification is applied by dripping one drop from each sample onto filter paper and keeping it in an oven at 100 °C for 10 min (Figure 3) [24].

### 4.5. GC-MS Analysis

Gas chromatography-mass spectrometry analyses were performed according to the Servi et al. [19] method. 20 μL PTEO samples were diluted in 180 μL n-hexane (GC grade, Sigma-Aldrich) prior to analysis. 1 µL of each diluted sample was injected into the GC injector (Agilent Technologies, Santa Clara, CA, USA) in split mode with a ratio of 1:50. The injector temperature was set to 250 °C. An HP-5MS column was used (5% diphenyl, 95% dimethyl polysiloxane; 30 m × 0.25 mm, 0.25 µm film thickness). The oven temperature was set to 60 °C for 1 min and then set to 246 °C to increase by 3 °C per minute. Afterwards, it was kept at this temperature for 30 min. The carrier gas was helium, and the flow rate was set to 0.9 mL per minute. The mass range was found to be 35–420 *m*/*z*, while the ion source temperature was set to 230 °C and the transfer line intake temperature was set at 250 °C. The determination of the components was decided by comparing the n-alkane series (C5 to C30) of previous studies with the RRI (relative retention indices) values of essential oils and comparing the mass spectra with the NIST14 and Wiley7 libraries, and the quantification of the percentages of the compounds was calculated via areas of the MS chromatograms. The same GC–MS chromatographic conditions were applied to all essential oil samples and the standard mixture.

## 5. Conclusions

Essential oils possess a rich chemical composition that allows them to be used as plant-based medicinal ingredients in prophylactic/therapeutic treatments in various industries, including pharmacy. Therefore, it is expected of them to comply with international standardization criteria to be regarded as high-quality products. Additionally, the fact that essential oils are on the market on many platforms other than community pharmacies indicates that restrictions are not fulfilled, which leads to the requirement of quality control. It is unwanted for products used in public health to pose such risk. Products marketed for health benefits are required to comply with officially recognized pharmacopoeia standards. It has been deemed necessary to carry out up-to-date quality analyses on the Turkish market according to the European Pharmacopoeia 10.0 due to being the first to conduct such a study in commercial PTEO control analyses in literature. None of the 14 samples fully met all the monograph standards. The A3 coded sample showed the least compatibility by meeting only 3 criteria out of 17, whereas A5, P1, and P4-coded samples showed overall more compliant results by meeting 11 criteria out of 17. Comparing the points of sale, it was calculated that the pharmacy-based pine turpentine essential oil samples showed more compatible results (59%) than the samples obtained from non-pharmacy sales points (49.5%). However, these rates were found to be quite low. The pronounced chemical variability observed may be attributed to the presence of heterogeneous mixtures comprising different, lower-cost turpentine sources employed for diverse industrial applications. This variability raises concerns regarding the accurate identification of authentic *Pinus* species and indicates that product labeling may inadequately represent the actual chemical composition. As adulteration practices are diverse and difficult to anticipate, quality control assessments of pine turpentine essential oils available on the Turkish market should be expanded through the integration of advanced adulteration-detection strategies.

## Figures and Tables

**Table 1:** Relative density, refractive index, and optical rotation assay results. Assays were performed in triplicate.

Samples	Relative Density (0.856 to 0.872)	Refractive Index (1.4650 to 1.4750)	Optical Rotation (−40° to −28°)
A1	0.892 ± 0.000	1.4764 ± 0.0000	5.32 ± 0.00
A2	0.864 ± 0.000	1.4724 ± 0.0001	19.64 ± 0.00
A3	0.789 ± 0.000	1.4733 ± 0.0000	6.35 ± 0.00
A4	0.868 ± 0.000	1.4712 ± 0.0000	−28.28 ± 0.00
A5	0.688 ± 0.001	1.4715 ± 0.0000	−36.52 ± 0.00
A6	0.843 ± 0.000	1.4770 ± 0.0001	−37.02 ± 0.00
A7	0.846 ± 0.000	1.4704 ± 0.0000	27.4 ± 0.00
A8	0.841 ± 0.001	1.4673 ± 0.0001	−33.48 ± 0.00
A9	0.849 ± 0.000	1.4693 ± 0.0000	−46.08 ± 0.00
A10	0.852 ± 0.000	1.4661 ± 0.0001	−30.74 ± 0.00
P1	0.863 ± 0.001	1.4719 ± 0.0000	−36.53 ± 0.00
P2	0.863 ± 0.000	1.4700 ± 0.0001	−29.97 ± 0.00
P3	0.871 ± 0.001	1.4712 ± 0.0000	−17.13 ± 0.00
P4	0.875 ± 0.001	1.4696 ± 0.0001	−29.44 ± 0.00

**Table 2:** Phytochemical profile of pine turpentine essential oil samples in relative percentage.

Compound	RT	RI^exp^	RI^lit^	RI^std^	P1	P2	P3	P4	A1	A2	A3	A4	A5	A6	A7	A8	A9	A10
Tricyclene	5.97	921	926													0.5	0.3	0.5
α-Thujene	6.09	925	927												2.2			
α-Pinene	6.52	939	936	938	41.9	35.8	40.2	51.8	3.5	31.8	37.4	57.9	51.8	30.2	35.7	38.3	50.3	39.3
Camphene	6.77	949	949		2.2	2.5	3.6	3.8	2.4	1.6	1.1	4.2	2.0	4.0	1.3	5.2	5.0	5.0
2,4-Thujadiene	6.91	953	956		0.1			0.1										
Sabinene	7.46	972	976												0.7			
β-Pinene	7.71	980	980		18.0	1.6	3.1	11.8	0.1	8.0	3.7	8.4	20.4	2.6	3.6	3.1	7.3	3.0
Myrcene	8.03	991	992	986	1.4	1.3	0.9	1.4	1.9	1.9	1.6	1.6	0.9	0.8	1.8	0.9	1.8	1.0
α-Phellandrene	8.48	1005	1006				0.4		0.3	0.3		0.5			0.3	0.5	0.6	0.4
3-Carene	8.86	1015	1012		0.1	0.3	3.6	6.0	17.7	28.1	36.1	0.3		0.2	33.5	0.2	1.8	0.2
α-Terpinene	8.90	1016	1017	1013						1.0						0.9		
2-Carene	8.91	1016	1014				0.7					1.2			0.7			0.8
1,4-Cineole	9.07	1021	1016						2.3					0.3				
p-Cymene	9.22	1025	1025		0.5	0.9	1.4	0.7	0.1	3.6		0.8	0.5	3.0	2.3	1.7	1.0	1.6
Limonene	9.39	1029	1037	1024	5.2	39.9	20.7	7.1	12.7	7.2	8.3	8.8	6.7	12.8	6.6	13.0	10.5	16.0
γ-Terpinene	10.45	1057	1059			0.3	0.5		3.6	1.5	1.3	0.7			1.5	0.7	1.0	0.6
Fenchone	11.62	1088	1088											0.8				
α-Terpinolene	11.64	1089	1094		1.3	1.3		0.7	15.7	7.3	6.5	7.7			6.9	3.1	9.5	2.7
α-Pinene oxide	12.03	1099	1095		0.3			1.8					1.4	0.8				
Fenchol	12.74	1116	1123		0.4	0.4	0.5	0.2	4.0	0.2		0.4		1.3		0.8	0.5	0.7
Alloocimene	13.31	1129	1131				0.5									0.9		0.8
Pinocarveol	13.77	1140	1139					0.7										
1-Terpinenol	13.83	1142	1141						7.1									
Camphor	13.95	1144	1145	1140										1.0		0.6		
β-Terpineol	14.02	1146	1148			2.2	1.9	1.7	4.5					3.1		1.6	0.6	1.6
endo-Borneol	14.93	1168	1167	1164	0.4	0.6	1.1	0.4	4.2			0.5		2.4		1.6	0.5	1.6
Terpinen-4-ol	15.39	1179	1178	1174		2.1	2.6	0.6	2.9	0.6	0.4			4.3	0.5	3.2	0.3	3.1
α-Terpineol	16.06	1194	1195	1189	2.6	4.2	4.9	0.8	10.7	1.2	0.5	3.8	1.1	8.3	0.6	5.5	4.6	5.5
Myrtenol	16.27	1199	1199		0.5			0.7										
γ-Terpineol	16.57	1206	1217						2.3								0.3	
Verbenone	16.78	1211	1205		0.1		0.4							0.9				0.4
Bornyl acetate	20.02	1286	1287	1282	0.6								0.3					
α-Cubebene	22.73	1350	1350		1.2	0.4		0.3					0.5					
Longicyclene	23.58	1370	1368		0.3													
α-Ylangene	23.64	1372	1372											0.7				0.4
α-Copaene	23.84	1376	1376		0.9	0.3	0.5	0.3					0.4	1.2		0.9		0.8
Longifolene	25.09	1406	1406		6.4	0.9	0.7	1.7	0.2			2.8	3.1	0.6		0.4	3.3	0.4
β-Caryophyllene	25.71	1421	1425	1412	7.6	2.1	1.0	0.6	0.1				1.7				0.2	0.4
α-Humulene	27.05	1455	1454		1.1	0.3		0.2					0.3					
γ-Muurolene	27.98	1478	1478		0.1	0.7	1.3	0.7						3.2		2.2		1.9
α-Muurolene	28.95	1501	1502		0.3		0.9	0.4						1.4		1.3		1.1
γ-Cadinene	29.49	1515	1515			0.4		0.4						1.9		1.4		1.2
δ-Cadinene	29.87	1525	1525		1.4	1.2	2.7	0.6						3.4		3.9		3.5
Cubenene	30.24	1535	1532															0.2
α-Cadinene	30.43	1540	1541											1.4				0.3
Caryophyllene oxide	32.19	1585	1581	1579	2.1			0.5					3.8					
Sandaracopimaral	51.55	2085	2185				0.7									1.1		
			Total (%)		97.1	99.7	94.9	95.3	96.3	94.2	96.9	99.7	94.6	90.6	98.3	92.9	99.1	94.5

RT: Retention time, RI^exp^: Retention index of the conducted experiment, RI^lit^: Retention index in literature, RI^std^: Retention index of reference/standard mixture.

## Data Availability

The raw data supporting the conclusions of this article will be made available by the authors on request.

## Abbreviations

PTEO	Pine turpentine essential oil
EP	European Pharmacopoeia
HPTLC	High performance thin-layer chromatography
GC-MS	Gas chromatography-mass spectrometry
[α]tλ	Optical rotation value
IA	Acid value
IP	Peroxide value
FID	Flame ionization detector
